# Mesh-based detailed skeletal models for the ICRP reference pediatric individuals: development and dosimetric implications

**DOI:** 10.1088/1361-6560/ae501a

**Published:** 2026-03-19

**Authors:** Chansoo Choi, Robert J Dawson, Bangho Shin, Yitian Wang, Johannes Tran-Gia, Maikol Salas Ramirez, Anna-Lena Theisen, Michael Lassmann, Wesley E Bolch

**Affiliations:** 1 J. Crayton Pruitt Family Department of Biomedical Engineering, University of Florida, Gainesville, FL, United States of America; 2 Medical Physics Program College of Medicine, University of Florida, Gainesville, FL, United States of America; 3 Department of Nuclear Medicine, University Hospital Würzburg, Würzburg, Germany

**Keywords:** skeletal dosimetry, ICRP reference pediatric individuals, mesh format, red bone marrow, endosteum

## Abstract

**Objective.:**

The skeleton contains the red bone marrow (RBM) and the endosteum, tissues linked to radiation-induced leukemia and bone cancer, making their consideration essential in radiation dosimetry. Although adult skeletal dosimetry has advanced with 3D images such as *μ*CT images, the scarcity of comparable pediatric images prevents pediatric skeletal dosimetry from achieving a similar level. This study aims to develop 3D image-based detailed pediatric skeletal models that, while grounded in adult *μ*CT images, incorporate the anatomical features of the developing pediatric skeleton.

**Approach.:**

Target skeletal values were established from extensive anatomical literature and International Commission on Radiological Protection publications, including skeletal tissue mass, cellularity factor, trabecular bone volume fraction, and trabecular number. Guided by these values, trabecular bone models converted from adult *μ*CT images were refined, a 50 *μ*m endosteal layer was defined, yellow bone marrow (YBM) was incorporated as adipocytes, and remaining regions were assigned as RBM. All modeling steps were performed automatically using our C++-based bone modeling program.

**Main results.:**

A total of 246 pediatric skeletal models were developed in a high-quality mesh format across six age and sex groups (sex-averaged newborn, 1 year-old, 5 year-old, and 10 year-old, and sex-specific 15 year-old male and female), with each group comprising 41 models. These models represent trabecular bone and RBM/YBM in both the shallow and deep marrow, and all matched their target values within 2%. For selected cases, PHITS Monte Carlo simulations were used to calculate specific absorbed fractions, which increased with decreasing age due to differences in target mass and the combined effects of the anatomical factors incorporated in this study.

**Significance.:**

This study provides the first comprehensive set of 3D image-based pediatric skeletal models for skeletal dosimetry. These models, together with the dosimetric datasets derived from them, are expected to provide an anatomically robust foundation for improving the accuracy and reliability of pediatric skeletal dosimetry.

## 1. Introduction

The skeleton, in which tissues vulnerable to radiation-induced health risks reside, is regarded as one of the most critical anatomical structures in radiation dosimetry. Among skeletal tissues, the red bone marrow (RBM) is of particular significance, as it houses hematopoietic stem cells responsible for blood cell production and can give rise to stochastic effects such as radiogenic leukemia following radiation exposure. Accordingly, the International Commission on Radiological Protection (ICRP) assigned the RBM the highest tissue-weighting factor of 0.12 among all tissues considered in the calculation of effective dose (ICRP 2007). In addition, exposure of the RBM to high-dose radiation over a short period can induce deterministic effects, including the hematopoietic subsyndrome of acute radiation syndrome (H-ARS), which manifests as symptoms ranging from malaise and fatigue to increased susceptibility to infection and, in severe cases, death (Strom 2003, Adams et al 2017). Compared with subsyndromes involving other tissues, H-ARS has the lowest threshold dose and is therefore the most frequently observed type. The endosteum, another skeletal tissue, contains osteoprogenitor cells required for bone growth, shaping, and maintenance, and can lead to stochastic effects such as radiogenic bone cancer following radiation exposure. The ICRP assigned a tissue-weighting factor of 0.01 to the endosteum (ICRP 2007) and defined it as a 50 *μ*m thick marrow layer lining the trabecular surfaces of the spongiosa and the inner cortical surfaces of the medullary cavity (Bolch et al 2007, ICRP 2009).

Despite their importance, skeletal dosimetry has remained a challenging task because these skeletal tissues are primarily distributed within the spongiosa, which has a highly complex microarchitecture. In recent years, the most advanced approach to skeletal dosimetry has involved integrating detailed skeletal models derived from 3D images such as micro-computed tomography (*μ*CT) images with whole-body computational phantoms and employing them in Monte Carlo radiation transport simulations. For example, the research group at the University of Florida (UF) acquired *μ*CT images from spongiosa samples taken from 32 skeletal sites of a 40 year-old male cadaver and 37 skeletal sites of a 45 year-old female cadaver to develop voxel-based detailed skeletal models (Hough et al 2011, O’Reilly et al 2016). In addition, research groups at the Federal University of Pernambuco and Tsinghua University developed voxel-based skeletal models using *μ*CT images obtained from eight skeletal sites of male and female cadavers and from 32 skeletal sites of a male cadaver, respectively (Kramer et al 2006, 2007, Gao et al 2017). Most recently, the UF research group developed mesh-based skeletal models (Choi et al 2025)—which are regarded as the most advanced modeling format in computational dosimetry (Kainz et al 2019)—using the same *μ*CT images employed in earlier studies (Hough et al 2011, O’Reilly et al 2016). Unlike voxel-based models, which are limited by staircase geometry and restricted flexibility, these mesh-based models provide a more realistic representation of skeletal tissues and are aligned with target skeletal values established from extensive anatomical literature and the ICRP Reference Adults (ICRP 2002, 2020a).

However, although extensive efforts have been devoted to developing 3D image-based detailed skeletal models for adults, very limited work has been conducted for children, who not only are more radiosensitive and have longer remaining lifespans (ICRP 2013, UNSCEAR 2013) but also possess a higher marrow cellularity (ICRP 1995). This lack of progress in pediatric skeletal dosimetry is most likely due to ethical considerations and the limited availability of pediatric cadaveric specimens. Pafundi et al (2009) developed, to our knowledge, the only 3D image-based detailed skeletal models, based on *μ*CT images of spongiosa samples obtained from available skeletal sites of 4 d-old and 5 d-old newborn cadavers. As a result, although the need for pediatric skeletal dosimetry has long been recognized, it still relies on values derived from limited datasets (Beddoe 1976, Pafundi 2009, Pafundi et al 2009) combined with simple interpolations and assumptions (ICRP 2020b, 2023), which may not adequately reflect the nonlinear, site-specific nature of skeletal tissue changes during growth.

In the present study, therefore, we developed 3D image-based detailed skeletal models for children across six age and sex groups—sex-averaged newborn, 1 year-old, 5 year-old, and 10 year-old, and sex-specific 15 year-old male and female—comprising 41 models per group and a total of 246 models, pioneering the application of this approach to pediatric skeletal dosimetry. These pediatric skeletal models were constructed to serve as representative models for the ICRP Reference Pediatric Individuals (ICRP 2002) and to be used in conjunction with the most recent whole-body computational pediatric phantoms described in ICRP Publication 156 (ICRP 2024), namely the pediatric mesh-type reference computational phantoms (MRCPs). Although adult *μ*CT images were used due to the aforementioned limitations in data acquisition, an updated version of our previously developed bone modeling program (Choi et al 2025) was applied to deform the models in accordance with age- and site-specific target skeletal values derived from extensive anatomical literature and the ICRP Reference Pediatric Individuals (ICRP 2002, 2024), thereby capturing the anatomical characteristics of the developing pediatric skeleton. In addition, to investigate their dosimetric impact with particular emphasis on age dependency, specific absorbed fractions (SAFs) were calculated for selected skeletal sites, including the sacrum and proximal humeri, and the resulting values were compared with those obtained for adults.

## 2. Materials and methods

### 2.1. Pediatric MRCPs of ICRP Publication 156

**Figure 1.**
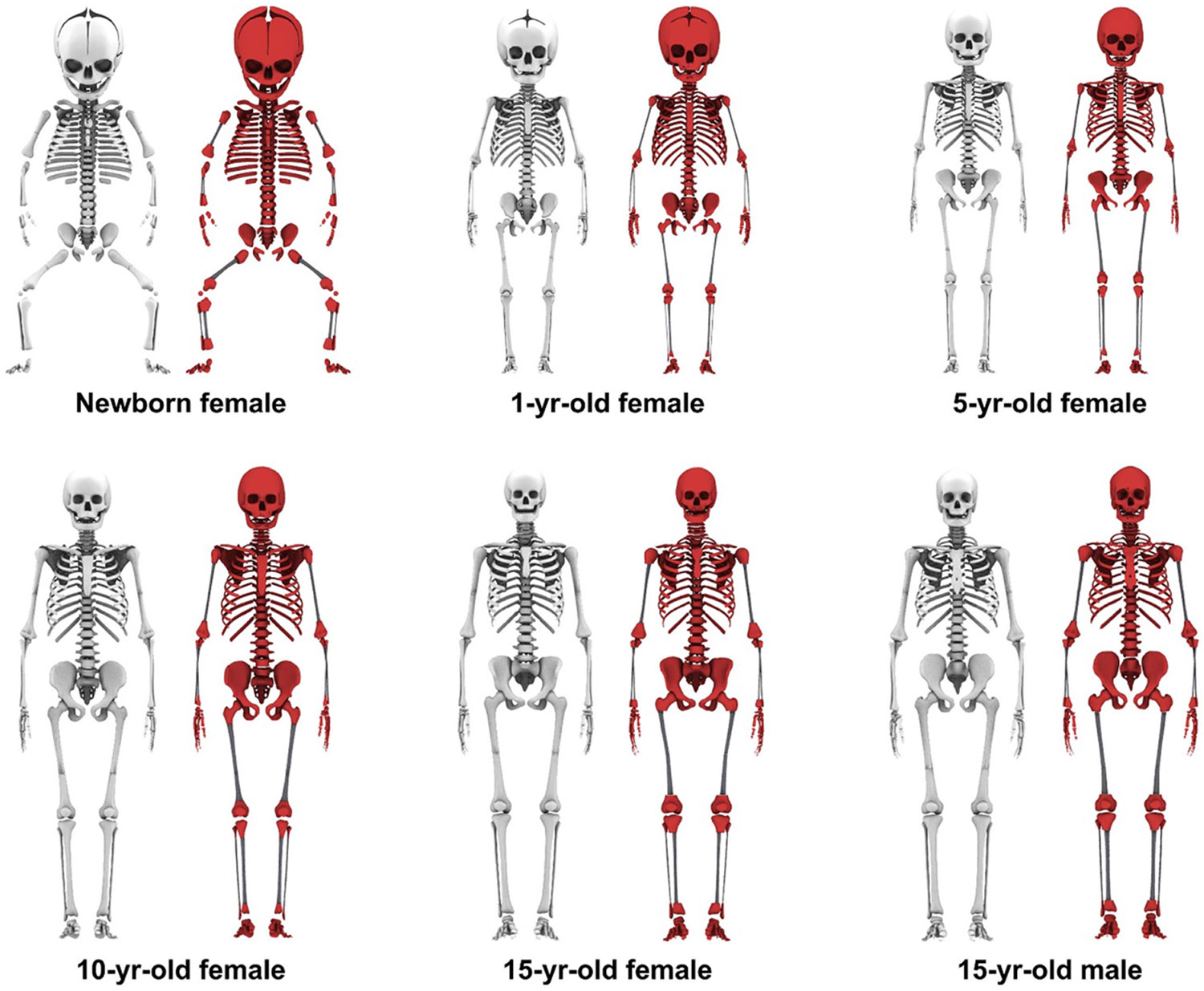
Skeletal systems of the pediatric mesh-type reference computational phantoms (MRCPs) in ICRP Publication 156 (ICRP 2024): cortical bone (white), spongiosa (red), and medullary cavity (black). For illustration purposes, only the female phantoms under 10 years of age are shown.

Figure 1 shows the skeletal systems of the pediatric MRCPs in ICRP Publication 156 (ICRP 2024), with which the detailed pediatric skeletal models developed in the present study will be used for skeletal dosimetry. The pediatric MRCPs consist of ten phantoms representing newborn, 1 year-old, 5 year-old, 10 year-old, and 15 year-old males and females, with identical skeletal systems for ages below 10 years. Most skeletal sites are subdivided into cortical bone and spongiosa, with the long bones additionally including a medullary cavity. The spongiosa, which possesses a highly complex microarchitecture, is represented as a single homogeneous region within each skeletal site under the assumption that the constituent skeletal tissues are uniformly mixed. The total masses of the skeletal tissues comprising these skeletal systems are aligned with the values for the ICRP Reference Pediatric Individuals provided in ICRP Publication 89 (ICRP 2002), except for the cortical bone and trabecular bone masses, which were modified according to the revised values reported by Pafundi (2009). In addition, their densities and elemental compositions are based on values provided in ICRP Publication 89 and ICRU Report 46 (1992), which reflect age-related mineral bone characteristics, including reduced mineralization, smaller and less perfect apatite crystals, and lower carbonation in younger individuals (Lefèvre et al 2019). Note that these total masses and material properties refer to the parenchymal tissues, while the skeletal systems are made consistent with these values after accounting for blood contents based on regional blood volume fractions (ICRP 2002) and for miscellaneous tissues distributed to the skeletal tissues according to their respective volume fractions (Pafundi 2009). Further details on the characteristics and data of the skeletal systems in the pediatric MRCPs can be found in Choi et al (2021). The detailed pediatric skeletal models developed in the present study are designed to explicitly represent the spongiosa within these skeletal systems across the six groups—sex-averaged newborn, 1 year-old, 5 year-old, and 10 year-old, and sex-specific 15 year-old male and female—with the goal of maintaining consistency in the total masses and material properties of the skeletal tissues.

### 2.2. UF *μ*CT-based primitive trabecular bone models

**Figure 2.**
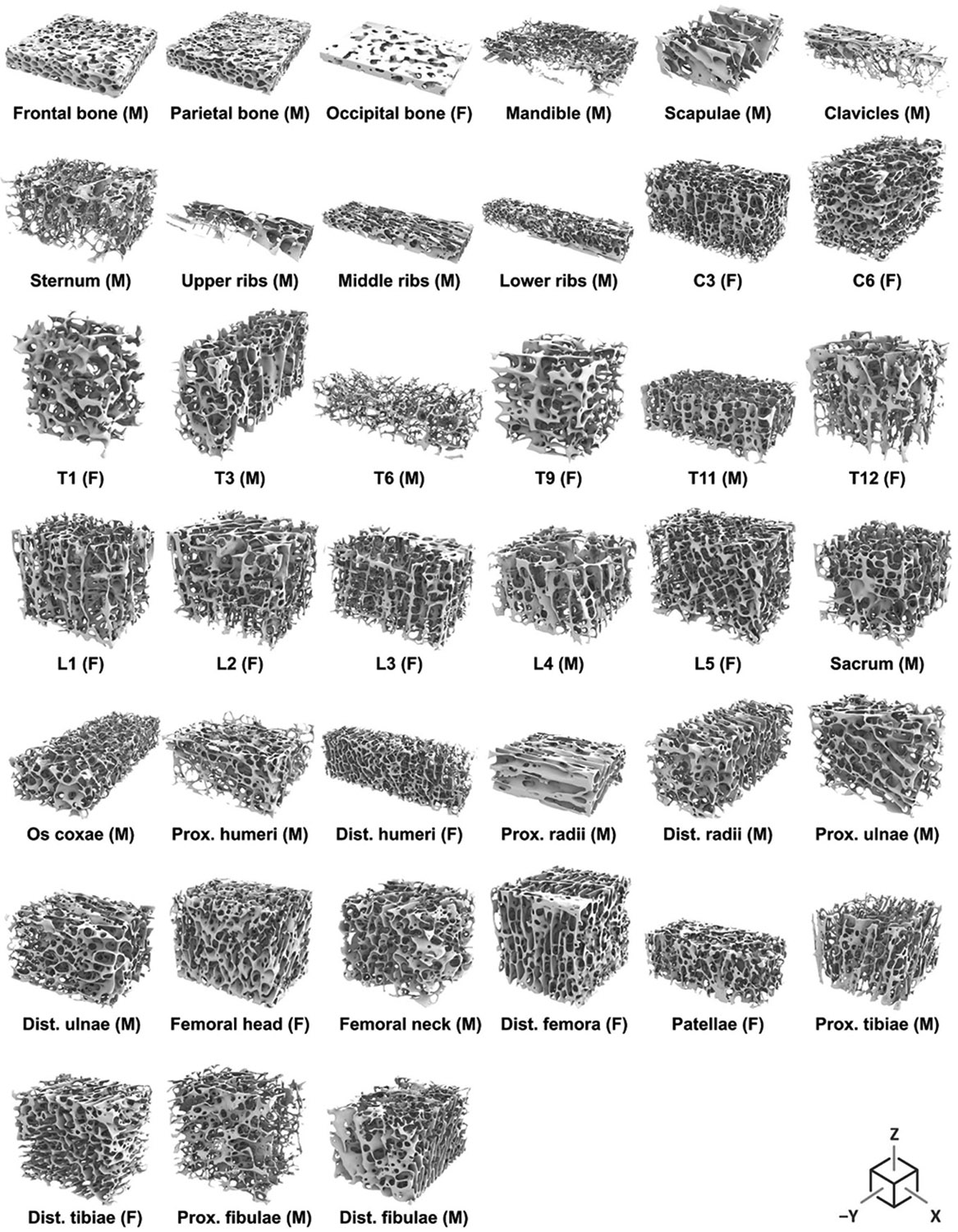
The 39 primitive trabecular bone models adopted in the present study for developing the detailed pediatric skeletal models. The letters in parentheses (M and F) denote models derived from adult male and adult female micro-computed tomography (*μ*CT) images, respectively.

In the present study, detailed pediatric skeletal models were developed based on the primitive trabecular bone models in the polygonal mesh format that were generated during the development of the adult skeletal models (Choi et al 2025), as shown in figure 2. These trabecular bone models were derived from UF *μ*CT images of adult spongiosa (Hough et al 2011, O’Reilly et al 2016), and table 1 summarizes information on these *μ*CT images, including the acquired skeletal sites and image dimensions (matrix size and total voxels). For the male, *μ*CT images were obtained from spongiosa samples collected from 32 skeletal sites of a 40 year-old cadaver (Hough et al 2011), whereas for the female, they were acquired from 37 skeletal sites of a 45 year-old cadaver (O’Reilly et al 2016). All images were acquired using a *μ*CT-80 system (Scanco Medical AG, Bassersdorf, Switzerland) operated at an isotropic voxel resolution of 30 *μ*m, and in these images, the trabecular bone and surrounding marrow regions could be clearly distinguished. In the present study, the adult male and female trabecular bone models were combined to establish a new subset of 39 trabecular bone models that served as the foundation for developing the detailed pediatric skeletal models. For skeletal sites available in only one sex, the corresponding model was adopted, whereas for sites present in both sexes, the model with the larger image dimensions was selected (see table 1).

### 2.3. Establishment of pediatric target skeletal values

To develop the detailed pediatric skeletal models based on the primitive trabecular bone models derived from adult *μ*CT images, the pediatric target skeletal values were first established. Because these trabecular bone models preserve adult trabecular morphology, they do not inherently reflect the site-specific morphological changes that occur in skeletal tissues during growth. In the present study, to capture the trabecular characteristics specific to children, two representative parameters of trabecular morphology—bone volume fraction (BV/TV) and trabecular number (Tb.N)—were considered. BV/TV represents the ratio of trabecular bone volume (TBV) to spongiosa volume within each skeletal site, whereas Tb.N represents the number of trabeculae per unit length (mm^−1^) within each site, corresponding to the inverse of the mean trabecular spacing and reflecting the degree of trabecular network connectivity. While BV/TV was also considered in the development of the adult skeletal models, Tb.N was newly incorporated in the present study to account for the rapid changes in trabecular connectivity associated with bone modeling and remodeling, particularly during early growth. Furthermore, to subdivide the region surrounding the trabecular bone, referred to as the marrow region, into RBM and yellow bone marrow (YBM), the cellularity factor (CF) was incorporated. CF represents the haematopoietically active volume within the marrow region, that is, the volume fraction of RBM relative to the marrow region. In addition, to ensure consistency with the ICRP Reference Pediatric Individuals (ICRP 2002) and ultimately achieve compatibility with the pediatric MRCPs (ICRP 2024), the total masses of skeletal tissues were also taken into account.

To establish target BV/TV and Tb.N values, a comprehensive anatomical literature review was conducted. Through this review, approximately 40 studies were identified, which, to our knowledge, represent the existing body of work providing BV/TV and/or Tb.N values for specific skeletal sites in children. From these studies, first, all available data were extracted to create a comprehensive dataset. When data for individual subjects were presented in tables, they were directly adopted. If such data were available only in graphical form, clearly identifiable data were digitized using the PlotDigitizer program (version 2.6.12, http://plotdigitizer.sourceforge.net). When only group-averaged data were available, they were adopted instead. Next, through systematic analysis of the comprehensive dataset, data involving identical or overlapping subjects and those containing apparent typographical errors were identified and excluded, resulting in a refined subset. Then, considering the age groups defined for the ICRP Reference Pediatric Individuals (newborn, 1 year, 5 years, 10 years, and 15 years), corresponding age bins were established, and the data within each bin of the refined subset were subsequently extracted and averaged. For the 5-, 10-, and 15 year-old groups, an age range of *±*1 year was adopted. In contrast, shorter age intervals were applied for the younger groups; the newborn group was set to span from 9 months of gestational age (−1 month) to 3 months postnatal age, whereas the 1 year-old group covered an age range of *±*3 months. The narrower negative range for the newborn group was deliberately selected to limit variability associated with rapid anatomical changes occurring prior to birth. During averaging, individual data were equally weighted (weight = 1), whereas group-averaged data were weighted by the reported number of subjects (weight = *n*). Note that sex differences were not considered for age groups below 10 years, whereas for the 15-year-old group, sex-specific values were adopted where available. In cases where multiple specimens were reported for the same skeletal site within a single subject, the corresponding values were averaged and assigned a single weight (weight = 1). Table 2 presents the 29 studies from which the BV/TV and Tb.N data used in the present study were derived for each age group and skeletal site.

Although BV/TV and Tb.N data were obtained through the aforementioned procedures, data for certain age groups or skeletal sites were unavailable due to limitations in the existing literature. To address these gaps, the following steps were employed. When data could be obtained by expanding the predefined age bins by a factor of two, they were adopted. When data were available for both younger and older age groups but missing for intermediate groups, values were linearly interpolated between the adjacent age groups. In cases where data for younger groups were available but those for older groups were lacking, additional literature was reviewed to obtain adult values (Ulrich et al 1999, Eckstein et al 2007, Drews et al 2008, Boruah et al 2015, Schmidt et al 2022, Schröder et al 2022), which were then interpolated linearly as a function of age (e.g. distal femora). For skeletal sites where data remained unavailable even after these steps, surrogate data were adopted from anatomically or functionally comparable skeletal sites (e.g. the proximal ulnae were replaced by the proximal tibiae). For isolated sites with limited anatomical correlation to other bones (e.g. clavicles or patellae), mean values averaged across all other skeletal sites were used to minimize potential dosimetric discrepancies. Consequently, BV/TV and Tb.N values were established for all age groups and skeletal sites. CF was adopted from ICRP Publication 70 (ICRP 1995) for all groups and sites.

Through these procedures, BV/TV, Tb.N, and CF values were obtained for all age groups and skeletal sites. However, if these data are directly applied to the detailed pediatric skeletal models and subsequently implemented within the pediatric MRCPs, discrepancies in the total masses of skeletal tissues inevitably arise. To eliminate these discrepancies, BV/TV and CF values were slightly scaled across all skeletal sites within each age group using uniform scaling factors (SFs). This approach was adopted to prevent excessive changes in specific skeletal sites. For example, for the 15 year-old male, BV/TV and CF values were increased by 27% and 4%, respectively, across all skeletal sites, yielding total masses consistent with those of the corresponding pediatric MRCPs. As Tb.N does not affect trabecular bone mass, it was retained as initially derived without further adjustment. The target Tb.N values can be achieved by scaling the trabecular bone models with reference to their original Tb.N values. For this purpose, the original Tb.N values were measured using the BoneJ software (Domander et al 2021), and the SF for each skeletal site was then calculated. Table 3 lists the final established target BV/TV, Tb.N (together with SFs), and CF values for the development of detailed pediatric skeletal models.

### 2.4. Development of detailed pediatric skeletal models

**Figure 3.**
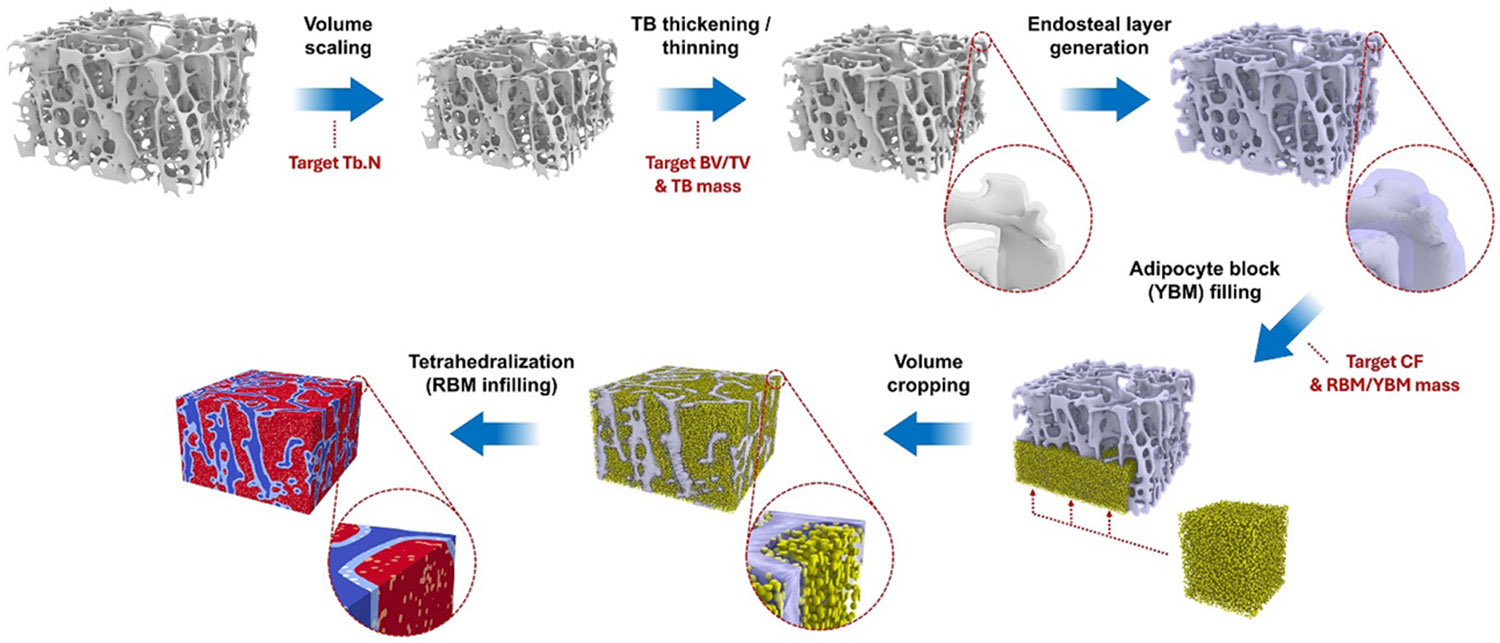
Schematic development procedure for developing the detailed pediatric skeletal models using the primitive trabecular bone models and the target skeletal values.

The detailed pediatric skeletal models were developed using the established target skeletal values and the primitive trabecular bone models in the polygonal mesh format. For this, an automated bone modeling program that was developed in our previous study for the adult skeletal models (Choi et al 2025) was updated and employed, and figure 3 presents the schematic development procedure using this program. Note that the program was written in C++ and implements several packages (e.g. the 3D Alpha Wrapping algorithm) of the Computational Geometry Algorithms Library (CGAL, www.cgal.org/).

The trabecular bone model was first scaled by applying the SF value to achieve the target Tb.N value, with this step being newly incorporated into the updated program. Next, the trabecular bone model was adjusted in thickness, being either increased or reduced to satisfy the target BV/TV value and, consequently, the target trabecular bone mass. An endosteal layer was then generated at a position 50 *μ*m from the trabecular surface. In addition, to prevent boundary ambiguity in subsequent steps, three auxiliary shell layers were created: one outward layer from the trabecular surface and both inward and outward layers from the endosteal surface, each with a 1 *μ*m offset.

Next, from a large library of pre-generated adipocyte (fat cell) block models, three candidates with CF values closest to the target were selected and repeatedly placed to fully occupy the entire cavity. Note that adipocytes, which constitute the majority of YBM, practically represent YBM, and their sizes follow an empirical Gaussian distribution reported by Rozman et al (1990). Using the trabecular shell layer, the trabecular bone region was subtracted from the placed block models of the three selected candidates, producing three corresponding YBM model versions. Among these, the one whose CF value most closely matched the target was selected. The selected YBM model was then subdivided using the endosteal shell layers into shallow (within the endosteum) and deep (outside the endosteum) regions, while the remaining space was defined as the RBM region, consisting of shallow and deep regions.

To ensure clear boundaries throughout the entire modeling procedure, a cropping factor was applied across all steps. For the adult skeletal models, this factor was fixed at 0.9, whereas in the pediatric models, the application of SF values frequently led to excessive model expansion. To maintain a consistent maximum model size, the cropping factor was therefore adjusted such that when the SF value exceeded 1, the default value of 0.9 was divided by the corresponding SF value. Finally, the developed models in the polygonal mesh format were tetrahedralized, completing the construction of the detailed pediatric skeletal models. Note that the tetrahedral mesh format is directly compatible with general-purpose Monte Carlo radiation transport codes such as PHITS (Sato et al 2024), Geant4 (Allison et al 2016), MCNP6 (Goorley et al 2013), and EGSnrc (Orok 2022), and provides excellent computational performance (Yeom et al 2014, 2019). Further technical details can be found in our previous study (Choi et al 2025).

### 2.5. Monte Carlo radiation transport simulations

To investigate the dosimetric impact of the detailed pediatric skeletal models, they were used, together with the previously developed adult models (Choi et al 2025), to calculate and compare electron SAFs in terms of age dependence. For this purpose, representative skeletal sites, i.e. the sacrum and proximal humeri, were selected, and radiation transport simulations were performed using the PHITS code (version 3.35) (Sato et al 2024). The source regions considered were the trabecular bone surface (TBS), TBV, RBM, and YBM, while the target regions were RBM and the endosteum. As in previous studies (O’Reilly et al 2016, Choi et al 2025), AFs, defined as the fraction of energy absorbed in a target region from that emitted by a source region, were calculated by combining microscale and macroscale dosimetry simulations using the following equation:

(1) $$\phi \;(r_{T}\leftarrow r_{S};E_{i},S_{j})=\phi_{\mu }\left(r_{T}\leftarrow r_{S};E_{i},S_{j}\right)\phi_{M}\left(\text{spongiosa}\leftarrow \text{spongiosa};E_{i},S_{j}\right)$$

where *ϕ_μ_* (*r_T_* ← *r_S_* *E_i_, S_j_*) is the AFs from the source region (*r*_S_) to the target region (*r*_T_), obtained through microscale dosimetry simulations performed under the assumption of an infinite spongiosa and calculated using the detailed skeletal models for a given energy (*E_i_*) and skeletal site (*S_j_*), while is the corresponding AFs obtained from macroscale dosimetry simulations using the spongiosa of the MRCPs, which allow the consideration of electron escape from the spongiosa. Subsequently, SAFs were calculated by dividing the simulated AFs by the mass of the corresponding target region. The simulations were performed on the UF’s HiPerGator 4.0 supercomputer (www.rc.ufl.edu/about/hipergator/), and the PHITS code environments configured for the microscale and macroscale dosimetry simulations are summarized in table 4. All simulations were performed such that the statistical relative errors in the target regions were maintained within 1%.

## 3. Results

### 3.1. Detailed pediatric skeletal models

**Figure 4.**
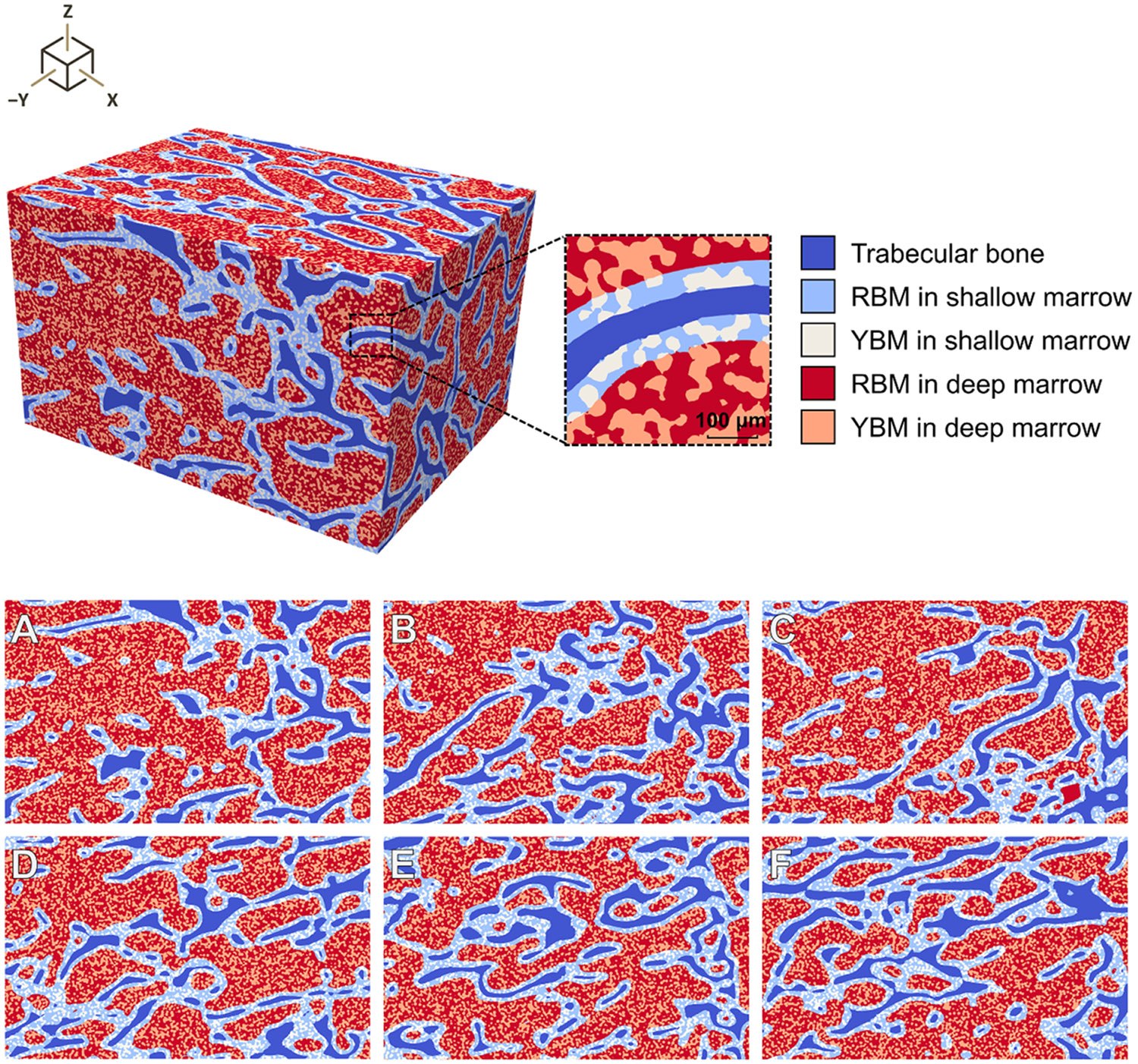
Detailed view of the 5 year-old distal ulnae model, showing six cross-sections along the *y*-axis from the −*y* surface (A) to the +*y* surface (F), with intermediate slices taken at equal intervals.

**Figure 5.**
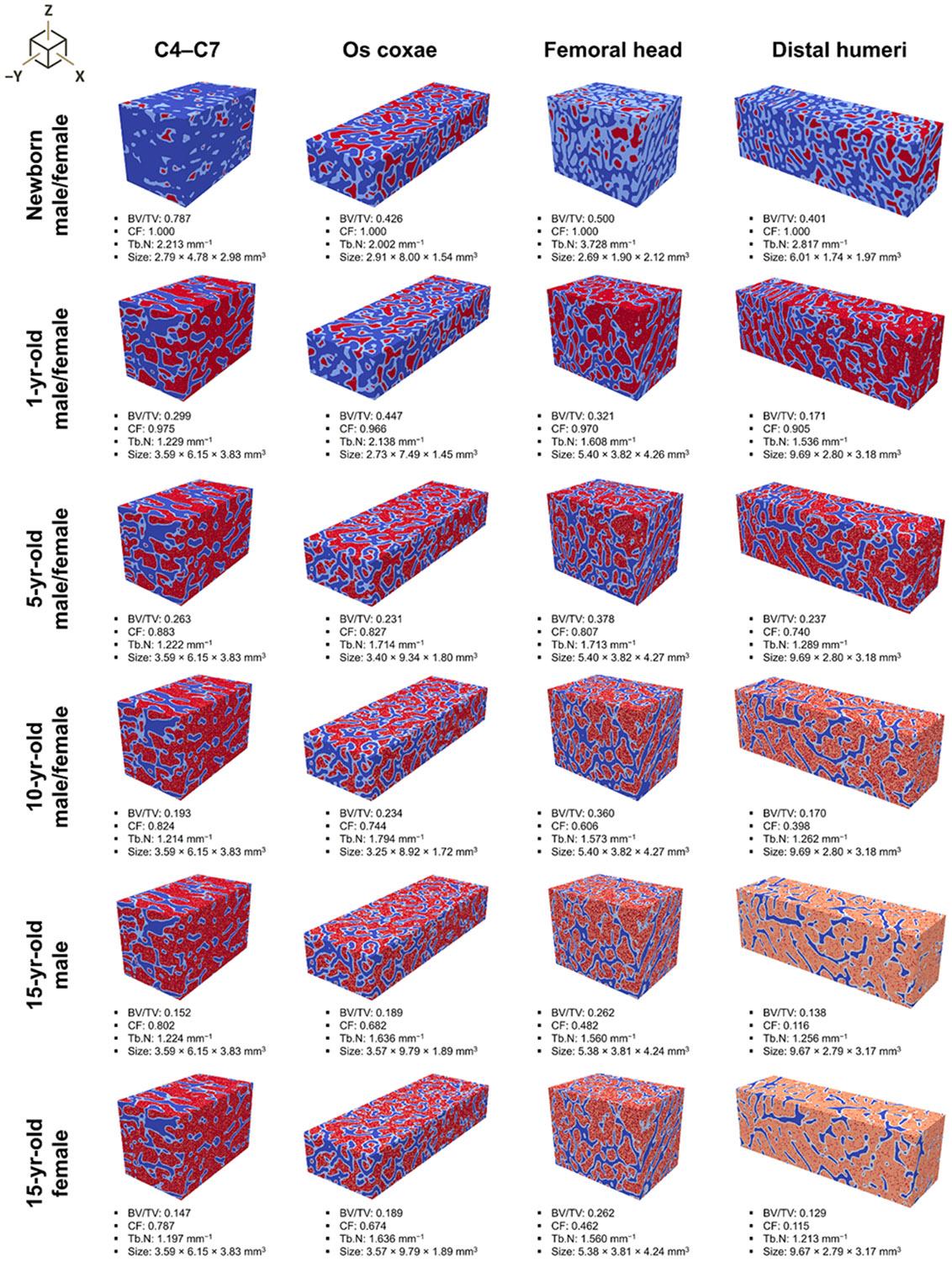
Overall views of the detailed skeletal models for the six groups across four selected skeletal sites.

In the present study, a total of 246 detailed pediatric skeletal models were developed across the six groups, including sex-averaged newborn, 1 year-old, 5 year-old, and 10 year-old, as well as sex-specific 15 year-old male and female, with 41 models for each group. Figure 4 shows a detailed view of the model for the 5 year-old distal ulnae as a representative example. As exemplified in this figure, most models consist of five distinct regions: trabecular bone, RBM and YBM within the endosteum (shallow marrow), and RBM and YBM beyond the endosteum (deep marrow). The 50 *μ*m thick endosteum is clearly represented overlying the trabecular bone. For the newborn models, in which all target CF values are unity, YBM is absent, while some 10- and 15 year-old models at distal skeletal sites (e.g. hand and foot bones) with target CF values of zero lack RBM, resulting in models composed of three regions instead of five. Figure 5 presents overall views of the models developed for the six groups, corresponding to the fourth to seventh cervical vertebrae, os coxae, femoral head, and distal humeri. Although the models for each skeletal site were developed from the same primitive trabecular bone models, all faithfully reproduce the age- and site-specific target skeletal values listed in table 3 within 2%, thereby clearly representing the anatomical characteristics of the developing pediatric skeleton.

Table 5 compares the total masses of skeletal tissues obtained after integrating the detailed pediatric skeletal models into the pediatric MRCPs (ICRP 2024) with the corresponding ICRP target masses (2002, 2024). Note that the target masses represent those of the parenchymal tissues with additional contributions from blood contents and miscellaneous tissues. As shown in this table, the values are in excellent agreement across all groups and skeletal tissues, with a maximum difference of only 1.6%. These results indicate that the developed models not only faithfully capture the anatomical characteristics of the pediatric skeleton but also remain consistent with the spongiosa of the pediatric MRCPs.

**Figure 6.**
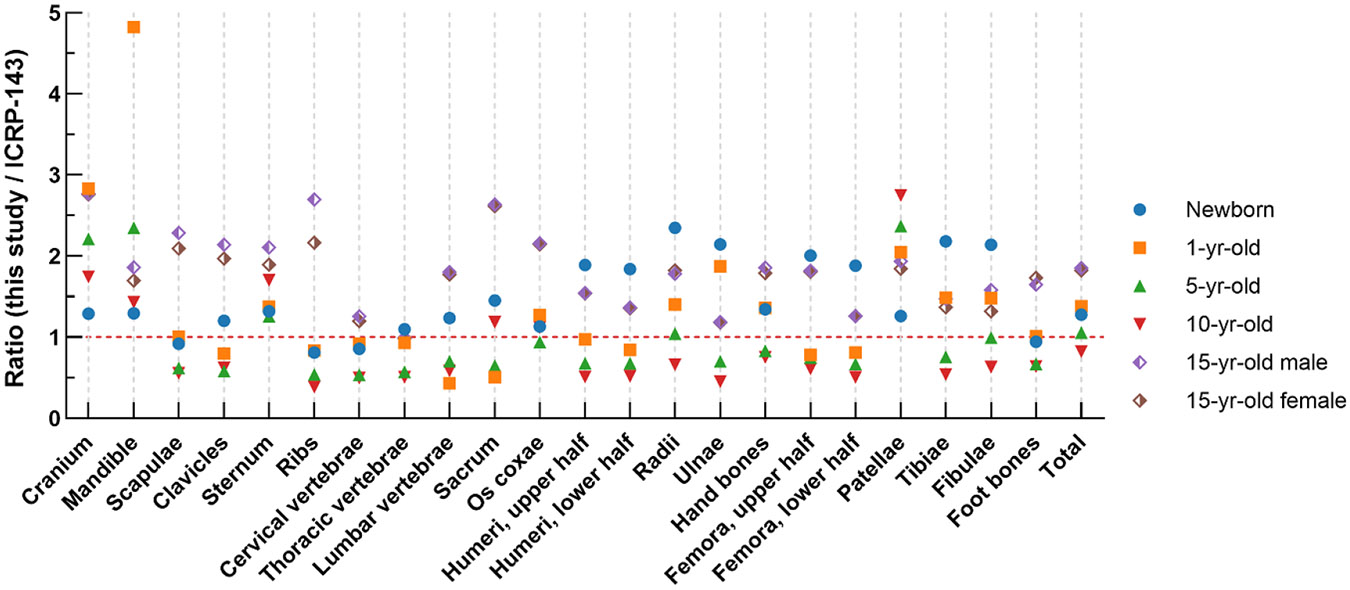
Ratio of endosteal masses derived in the present study to those reported in ICRP Publication 143 (ICRP 2020c).

Table 6 presents the dataset of masses for the target regions, RBM and endosteum, across age groups and skeletal sites, which are essential for skeletal dosimetry. To provide a complete dataset, the masses of the target regions within the medullary cavity, beyond the spongiosa considered in the present study, were also calculated from the pediatric MRCPs and included in this table. Figure 6 compares the endosteal masses newly derived in the present study with those provided in ICRP Publication 143 (ICRP 2020c), which have been used in pediatric skeletal dosimetry. Overall, significant differences were observed across all age groups and skeletal sites, with the largest difference, a factor of 4.8, found for the 1 year-old mandible. Such differences were also observed in the total endosteal masses, which were larger in the present study for all age groups except the 10-year-old group. The largest difference, a factor of 1.9, was found for the 15 year-old male group. Given that the previously reported endosteal masses (ICRP 2020c) were derived from limited pediatric data using simple estimation, the newly derived values in the present study provide a more reliable and accurate basis for pediatric skeletal dosimetry.

### 3.2. Dosimetric comparison

**Figure 7.**
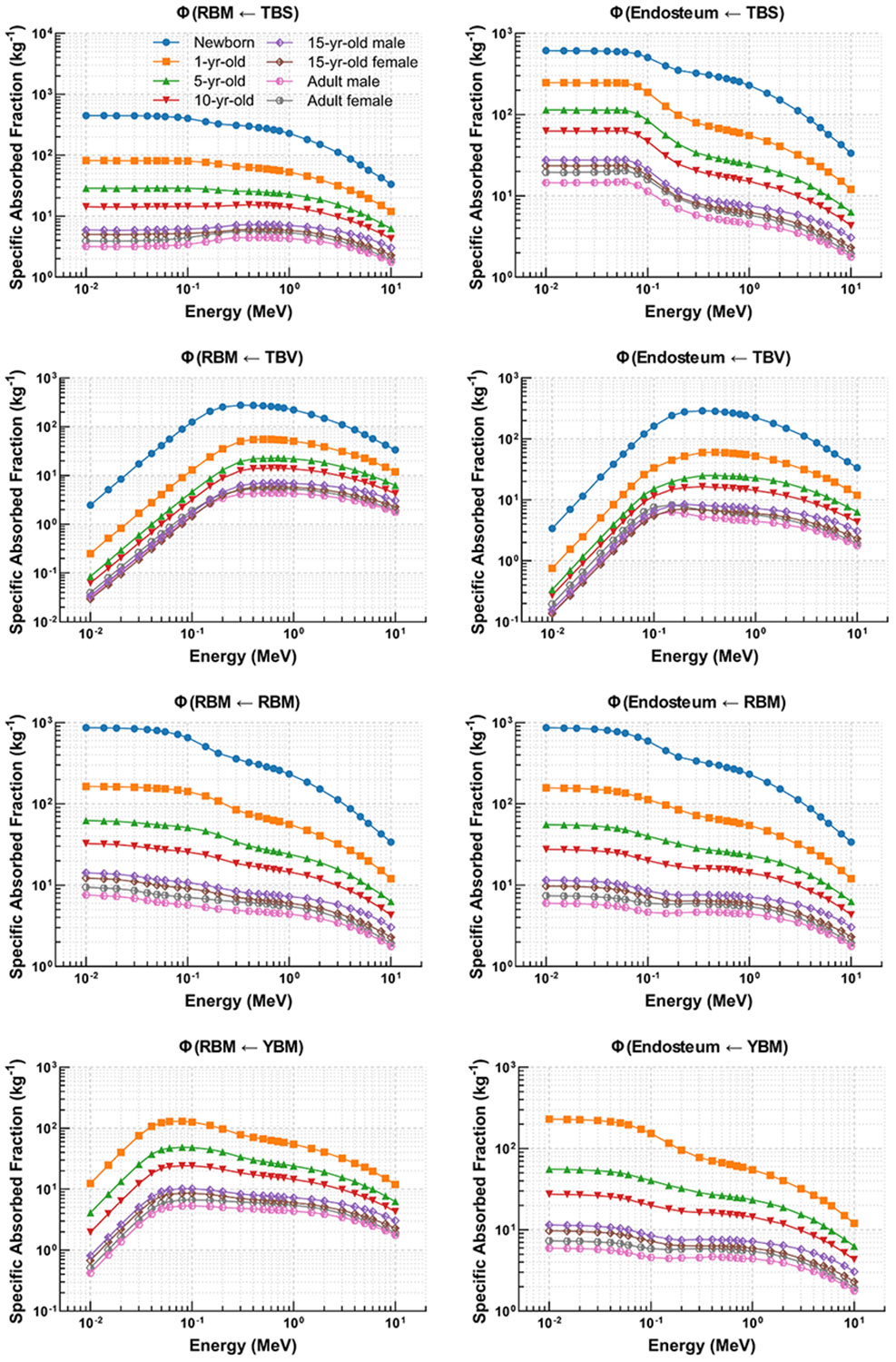
Electron specific absorbed fractions (SAFs) calculated for the sacrum, with the trabecular bone surface (TBS), trabecular bone volume (TBV), red bone marrow (RBM), and yellow bone marrow (YBM) as source regions and the RBM (left) and endosteum (right) as target regions.

Figure 7 shows electron SAFs calculated for the sacrum, comparing the six pediatric groups developed in the present study with the two adult groups of Choi et al (2025), where TBS, TBV, RBM, and YBM serve as the source regions and RBM and endosteum serve as the target regions. Note that YBM does not exist in the newborn group; therefore, no results appear for YBM as a source in this age group. Across all source-target combinations, a clear age-dependent trend is observed, with SAFs increasing as age decreases, driven primarily by reductions in target masses associated with the smaller skeletal sizes of younger groups (see table 6). Note that no distinct sex-specific trends are observed between the male and female groups of the same age, likely because the anatomical differences between them are relatively small. Although differences in target masses are the dominant contributor to these trends, other anatomical characteristics also play substantial roles.

**Figure 8.**
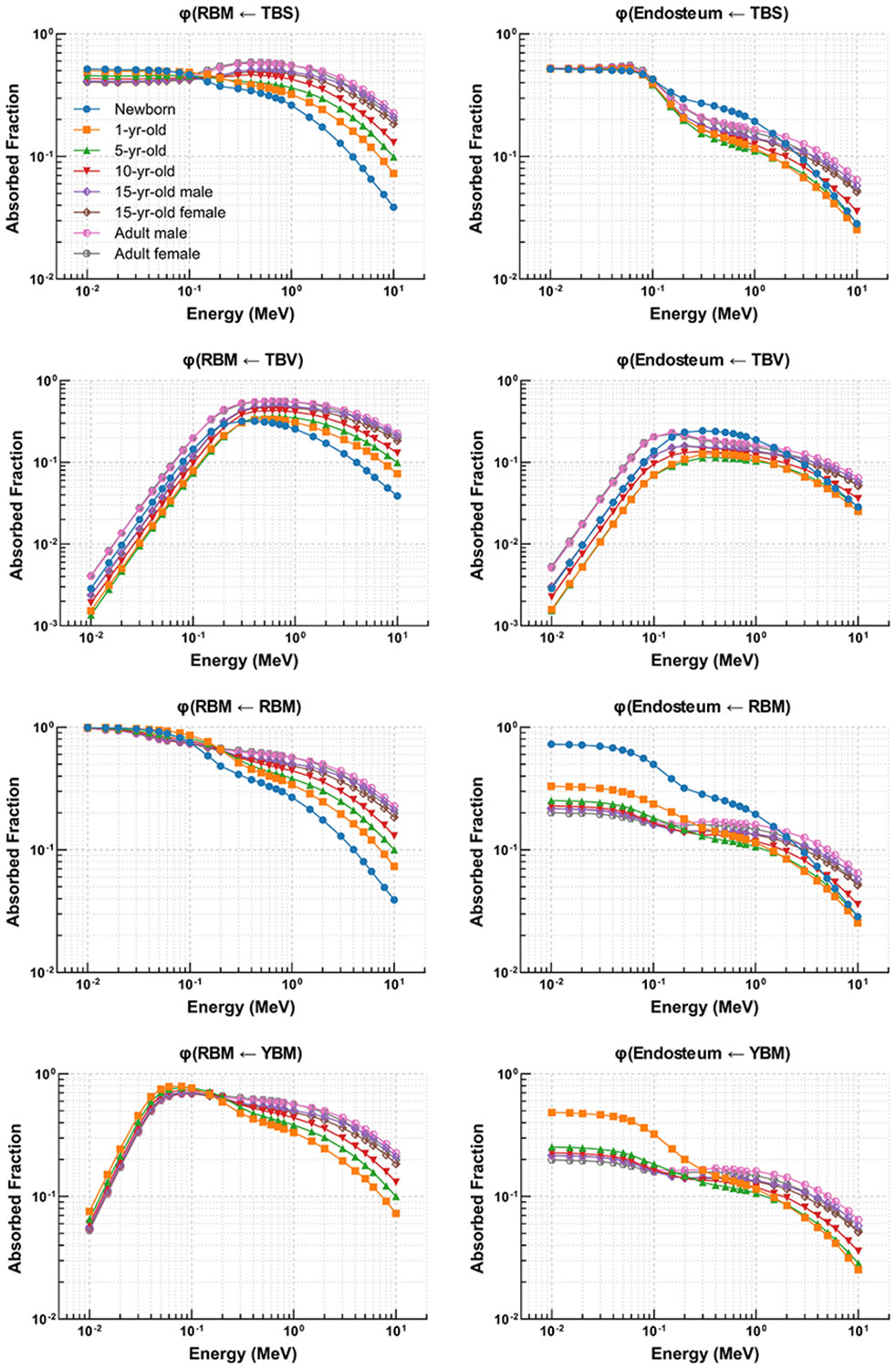
Electron absorbed fractions (AFs) calculated for the sacrum, with the trabecular bone surface (TBS), trabecular bone volume (TBV), red bone marrow (RBM), and yellow bone marrow (YBM) as source regions and the RBM (left) and endosteum (right) as target regions.

To clarify the influence of these additional factors, figure 8 presents the corresponding electron AFs. Even without the contribution of target masses, substantial age-dependent differences are still evident. For example, when TBS is the source and RBM the target, AFs at low energies increase with decreasing age because younger groups have larger CF values (see table 3), meaning that RBM occupies a greater fraction of the marrow region and therefore receives a larger proportion of the deposited energy. In contrast, when TBV is the source and RBM the target, AFs at low energies do not follow a monotonic age-related pattern. This occurs because, although CF values are higher at younger groups, BV/TV values are also higher (see table 3), increasing the fraction of energy deposited within the trabecular bone itself; these opposing effects act simultaneously. Another example is seen when RBM is the source and the endosteum the target. At low energies, AFs increase with decreasing age due to the higher BV/TV values in younger groups. The higher BV/TV values reduce the fraction of the spongiosa occupied by marrow, and since the endosteum thickness remains fixed at 50 *μ*m regardless of age, the endosteum occupies a relatively larger fraction of the marrow region in younger groups, thereby receiving a greater fraction of the deposited energy. At higher energies, AFs decrease for all age groups, but the decline is considerably steeper in younger groups due to the substantially greater particle escape associated with their smaller spongiosa volumes.

**Figure 9.**
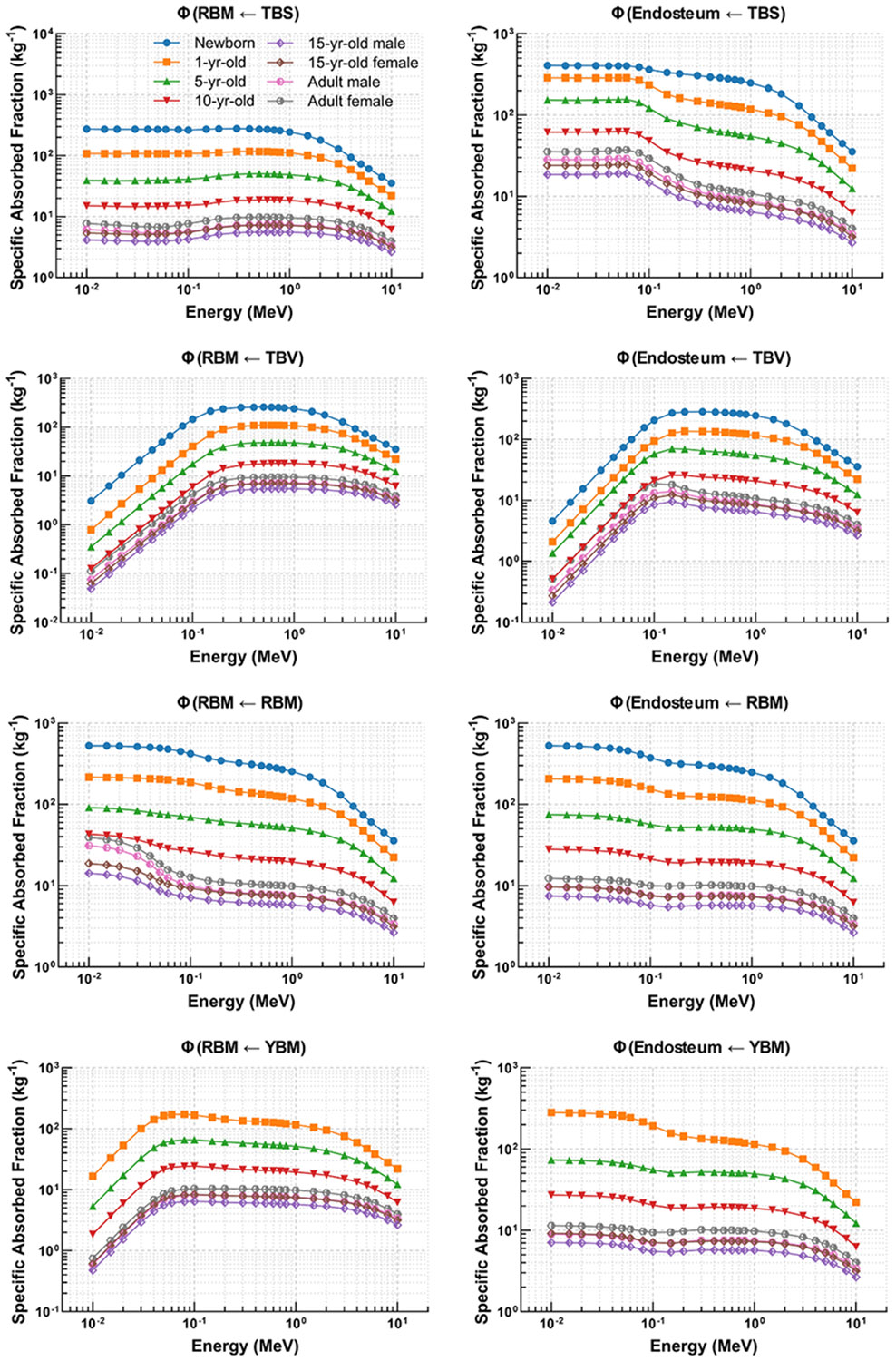
Electron specific absorbed fractions (SAFs) calculated for the proximal humeri, with the trabecular bone surface (TBS), trabecular bone volume (TBV), red bone marrow (RBM), and yellow bone marrow (YBM) as source regions and the RBM (left) and endosteum (right) as target regions.

**Figure 10.**
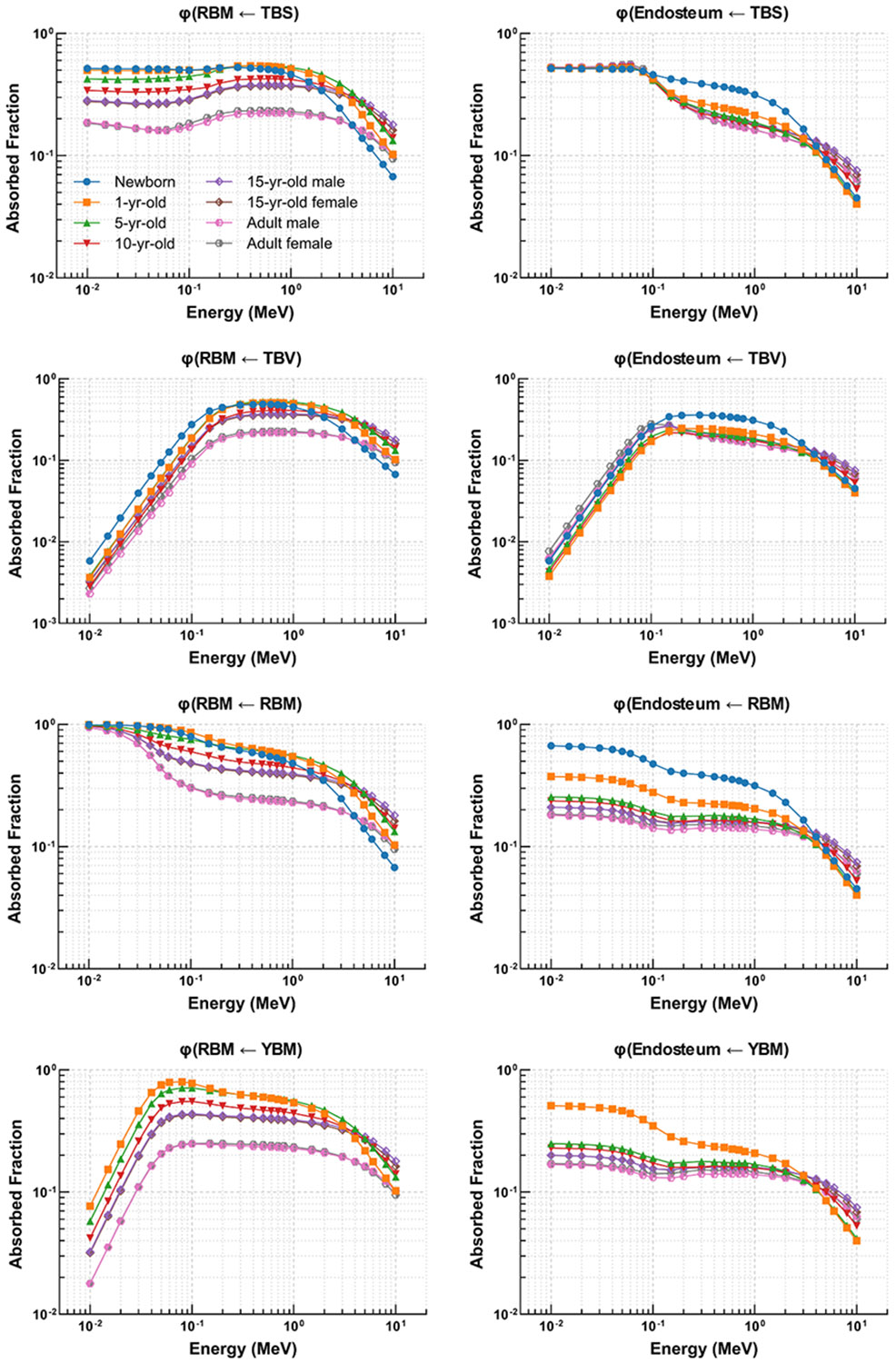
Electron absorbed fractions (AFs) calculated for the proximal humeri, with the trabecular bone surface (TBS), trabecular bone volume (TBV), red bone marrow (RBM), and yellow bone marrow (YBM) as source regions and the RBM (left) and endosteum (right) as target regions.

Figure 9 shows electron SAFs for the proximal humeri, and figure 10 presents the corresponding electron AFs. These figures show that age-dependent trends similar to those observed for the sacrum also appear for the proximal humeri. However, the extent of these differences varies between the two skeletal sites, not only because of differences in target masses but also due to distinct anatomical characteristics. In the proximal humeri, the range of CF values across the groups is larger (0.27–1.00) than in the sacrum (0.76–1.00), whereas the range of BV/TV values is smaller (0.07–0.34 in the proximal humeri vs 0.11–0.55 in the sacrum).

These comparative analyses show that SAFs are determined by the combined influence of age- and site-dependent differences not only in target masses but also in intrinsic anatomical characteristics. This indicates that pediatric skeletal dosimetry cannot be addressed by simple age-based scaling alone and instead requires models capable of accurately capturing the nonlinear and site-specific patterns of skeletal growth. We believe that the SAFs derived from the anatomically detailed pediatric skeletal models developed in the present study faithfully capture these complex age- and site-dependent variations and therefore provide a more reliable and anatomically grounded basis for pediatric skeletal dosimetry.

## 4. Discussion

The detailed pediatric skeletal models developed in the present study represent, to the best of our knowledge, the first comprehensive set of models constructed from 3D images specifically for pediatric skeletal dosimetry. However, several limitations remain. First, although considerable effort was made to faithfully capture various skeletal characteristics expected to be most relevant to skeletal dosimetry, including the CF, BV/TV, and Tb.N values, the models are based on adult *μ*CT images rather than pediatric images and therefore cannot fully reproduce the complete anatomical characteristics of the developing pediatric skeleton. As discussed earlier, this constraint stems from ethical considerations and the extremely limited availability of pediatric cadaveric specimens, leaving no practical alternative at present. To further enhance the accuracy and reliability of pediatric skeletal dosimetry, future work may explore the incorporation of other skeletal parameters, such as the degree of anisotropy and the structure model index. Although these parameters are expected to exert smaller dosimetric effects than those considered in the present study, their inclusion in the current modeling process could still offer meaningful improvements. Alternatively, if pediatric *μ*CT images become available in the future, even if limited to specific ages or skeletal sites, they could be incorporated through the same modeling process to replace the corresponding models in the present model set.

Second, although the models were developed to represent skeletal microstructure with high anatomical fidelity, this level of detail inevitably led to very large tetrahedral counts and correspondingly large file sizes. For example, the 5 year-old distal tibiae model contains approximately 200 million tetrahedra and has a file size of about 11 GB. While dosimetry simulations for individual skeletal sites are feasible, loading the full set of models for whole-body simulations would require several to tens of terabytes of RAM, as described in our previous study (Choi et al 2025). Such computational demands make whole-body applications impractical in typical computing environments. To address this limitation and enhance the usability of the model set, we plan to compute complete AF and SAF datasets for all age groups, skeletal sites, and particle types, and to use these datasets to develop a comprehensive dataset of pediatric fluence-to-dose response functions (DRFs) for photons. The photon DRFs currently provided in ICRP Publication 155 (ICRP 2023) were derived from limited pediatric datasets supplemented with simple interpolations and assumptions and therefore may not adequately reflect the nonlinear and site-specific patterns of skeletal development. In contrast, DRFs generated using the models of the present study will incorporate these age- and site-dependent anatomical characteristics. As a result, users will be able to perform accurate and reliable whole-body skeletal dosimetry without the need to install or manage the large model files directly.

Taken together, the methodological developments presented here provide a more anatomically robust foundation for pediatric skeletal dosimetry and will enable more reliable dose assessments. Given that RBM and endosteum are highly radiosensitive, and that children exhibit a greater lifetime susceptibility to stochastic radiation effects, maintaining high dosimetric accuracy is of particular importance in pediatric contexts. Although quantifying the clinical impact lies outside the scope of the present work, the models developed here, together with the dosimetric datasets that will be produced in future studies, can be integrated into patient-specific or cohort-based dosimetry frameworks to clarify how enhanced skeletal microstructure modeling influences dose estimates across pediatric applications—from radiological protection in diagnostic imaging to targeted radionuclide therapies such as systemic ^131^I-mIBG treatment for neuroblastoma (Gear et al 2020).

## 5. Conclusion

In the present study, we developed the first comprehensive set of 3D image-based detailed pediatric skeletal models for pediatric skeletal dosimetry, comprising a total of 246 models across six age and sex groups. These models were constructed by using primitive trabecular bone models derived from adult *μ*CT images as the starting point, adjusting the trabecular bone structures and defining the remaining skeletal tissues so that they match age- and site-specific target skeletal values established from extensive anatomical literature and from the ICRP Reference Pediatric Individuals (ICRP 2002, 2024). To investigate their dosimetric impact, electron SAFs were calculated using the developed models together with the pediatric MRCPs (ICRP 2024) for two representative skeletal sites, the sacrum and the proximal humeri. The resulting values were then compared across all age groups, including adults. The analyses revealed clear age-dependent trends, with SAFs increasing as age decreases, that were driven primarily by differences in target masses. Additional anatomical parameters incorporated into the model construction, including the BV/TV and CF values, were also found to exert substantial combined influences on SAFs. The models developed in the present study will be further utilized to compute AF and SAF datasets for a range of particle types and to generate a new DRF dataset. These forthcoming datasets are expected to markedly improve the accuracy and reliability of pediatric skeletal dosimetry and to provide a more anatomically robust foundation for future radiological protection and dosimetric applications.

## Figures and Tables

**Table 1.** Information on skeletal sites and image dimensions (matrix size in *x, y*, and *z* directions, and total voxel count) for the adult micro-computed tomography (*μ*CT) images from the University of Florida (UF) (Hough et al 2011, O’Reilly et al 2016). The *μ*CT images adopted in the present study are indicated. All images have an isotropic voxel resolution of 30 *μ*m.

Skeletal site	40-yr-old male		45-yr-old female		Selected images
	Matrix size	Total voxels	Matrix size	Total voxels	
Frontal bone	277 × 311 × 51	4393 497	132 × 103 × 17	231 132	Male
Parietal bone	249 × 358 × 49	4367 958	88 × 86 × 17	128 656	Male
Occipital bone	228 × 234 × 81	4321 512	161 × 125 × 12	241 500	Female^a^
Mandible	333 × 223 × 59	4381 281	112 × 76 × 56	476 672	Male
Scapulae	164 × 228 × 81	3028 752	90 × 103 × 65	602 550	Male
Clavicles	433 × 96 × 106	4406 208	277 × 177 × 86	4216 494	Male
Sternum	220 × 177 × 112	4361 280	144 × 142 × 100	2044 800	Male
Upper ribs	388 × 138 × 44	2355 936	84 × 39 × 37	121 212	Male
Middle ribs	494 × 169 × 53	4424 758	—	—	Male
Lower ribs	495 × 108 × 72	3849 120	96 × 29 × 28	77 952	Male
C3	109 × 90 × 75	735 750	249 × 134 × 131	4370 946	Female
C6	358 × 162 × 67	3885 732	133 × 228 × 142	4306 008	Female
T1	—	—	81 × 63 × 87	443 961	Female
T3	88 × 311 × 158	4324 144	106 × 100 × 91	964 600	Male
T6	338 × 144 × 90	4380 480	100 × 111 × 106	1176 600	Male
T9	—	—	109 × 109 × 102	1211 862	Female
T11	261 × 177 × 93	4296 321	—	—	Male
T12	—	—	159 × 123 × 137	2679 309	Female
L1	—	—	183 × 148 × 159	4306 356	Female
L2	167 × 232 × 112	4339 328	183 × 161 × 148	4360 524	Female
L3	—	—	217 × 148 × 136	4367 776	Female
L4	189 × 193 × 120	4377 240	171 × 183 × 138	4318 434	Male
L5	—	—	202 × 136 × 158	4340 576	Female
Sacrum	166 × 181 × 144	4326 624	140 × 85 × 105	1249 500	Male
Os coxae	144 × 396 × 76	4333 824	136 × 103 × 69	966 552	Male
Proximal humeri	214 × 218 × 94	4385 288	194 × 134 × 127	3301 492	Male
Distal humeri	141 × 132 × 235	4373 820	359 × 104 × 118	4405 648	Female
Proximal radii	229 × 176 × 87	3506 448	110 × 120 × 105	1386 000	Male
Distal radii	124 × 300 × 118	4389 600	223 × 183 × 107	4366 563	Male
Proximal ulnae	213 × 125 × 164	4366 500	180 × 95 × 120	2052 000	Male
Distal ulnae	201 × 153 × 123	3782 619	189 × 152 × 95	2729 160	Male
Femoral head	151 × 191 × 150	4326 150	199 × 141 × 157	4405 263	Female
Femoral neck	178 × 163 × 149	4323 086	204 × 42 × 77	659 736	Male
Distal femora	209 × 210 × 99	4345 110	158 × 171 × 162	4376 916	Female
Patellae	—	—	204 × 103 × 81	1701 972	Female
Proximal tibiae	175 × 182 × 136	4331 600	155 × 146 × 189	4277 070	Male
Distal tibiae	185 × 241 × 98	4369 330	189 × 142 × 164	4401 432	Female
Proximal fibulae	188 × 136 × 172	4397 696	209 × 115 × 115	2764 025	Male
Distal fibulae	155 × 213 × 132	4357 980	296 × 153 × 96	4347 648	Male

a Exceptionally, female *μ*CT images with a smaller total voxel count were adopted based on anatomical review, as the corresponding male images exhibited an unusually high bone volume fraction (BV/TV) exceeding 0.9, indicating a very limited marrow region, and were therefore not considered representative of the typical occipital spongiosa.

**Table 2.** List of references providing the adopted data used to establish the target bone volume fraction (BV/TV) and trabecular number (Tb.N) for each skeletal site and each age group (newborn, 1 year, 5 years, 10 years, and 15 years).

Skeletal site	Newborn	1 year	5 years	10 years	15 years
Parietal bone	Rodriguez-Florez et al (2017) ^a^	—	—	—	—
Scapulae	O’Malley (2013) ^b^	—	—	—	—
Sternum	Pafundi et al (2009) ^a^	—	—	—	—
Ribs	Beresheim et al (2020) ^b^ Byers et al (2000) ^b^ Pafundi et al (2009) ^a^	Beresheim et al (2020) ^b^ Byers et al (2000) ^b^	Beresheim et al (2020) ^b^ Byers et al (2000) ^b^	Beresheim et al (2020) ^b^ Byers et al (2000) ^b^	Beresheim et al (2020) ^b^
Cervical vertebrae	Acquaah et al (2015) ^b^ Pafundi et al (2009) ^a^	Acquaah et al (2015) ^b^	—	—	—
Thoracic vertebrae	Acquaah et al (2015) ^b^ Pafundi et al (2009) ^a^	Acquaah et al (2015) ^b^	—	—	—
Lumbar vertebrae	Acquaah et al (2015) ^b^ Pafundi et al (2009) ^a^	Acquaah et al (2015) ^b^	Kneissel et al (1997) ^b^	—	Kneissel et al (1997) ^b^
Sacrum	Yusof (2013) ^b^	Yusof (2013) ^b^	Yusof (2013) ^b^	—	—
Os coxae	Cunningham and Black (2009) ^b^ Pafundi et al (2009) ^a^ Volpato (2008) ^a^	Glorieux et al (2000)^b,c^ Parfitt et al (2000)^b,c^ Rauch (2012)^b,c^ Volpato (2008)^a^	Glorieux et al (2000) ^b,c^ Parfitt et al (2000) ^b,c^ Rauch (2012) ^b,c^	Glorieux et al (2000) ^b,c^ Parfitt et al (2000) ^b,c^ Rauch (2012) ^b,c^	Glorieux et al (2000) ^b,c^ Parfitt et al (2000) ^b,c^ Rauch (2012) ^b,c^
Proximal humeri	Chevalier et al (2021) ^b^ Colombo et al (2021) ^a^	Chevalier et al (2021) ^b^ Ryan et al (2017) ^b^	Chevalier et al (2021)^b^ Colombo et al (2021)^a^ Ryan et al (2017)^b^	Chevalier et al (2021) ^b^ Ryan et al (2017) ^b^	Chevalier et al (2021) ^b^
Skeletal site	Newborn	1 year	5 years	10 years	15 years
Distal radii	—	—	—	Kirmani et al (2009) ^b^ Mitchell et al (2018) ^b^	Chevalley et al (2011)^b^ Gabel et al (2017a)^b^ Kirmani et al (2009)^b^ Mitchell et al (2018)^b^
Proximal femora	Milovanovic et al (2017)^b^ Ryan and Krovitz (2006)^b^ Salle et al (2002)^b^	Milovanovic et al (2017) ^b^ Ryan and Krovitz (2006) ^b^ Ryan et al (2017) ^b^	Milovanovic et al (2017) ^b^ Ryan and Krovitz (2006) ^b^ Ryan et al (2017) ^b^	Milovanovic et al (2017) ^b^ Ryan and Krovitz (2006) ^b^ Ryan et al (2017) ^b^	—
Distal femora	Benjavongkulchai (2023) ^b^	Benjavongkulchai (2023) ^b^	Benjavongkulchai (2023) ^b^	—	—
Proximal tibiae	Benjavongkulchai (2023) ^b^ Gosman and Ketcham (2009) ^b^	Gosman and Ketcham (2009)	^b^ Benjavongkulchai (2023)^b^ Gosman and Ketcham (2009)^b^	Goliath (2017) ^b^ Gosman and Ketcham (2009) ^b^	Goliath (2017) ^b^ Gosman and Ketcham (2009) ^b^
Distal tibiae	—	—	—	Mitchell et al (2018) ^b^	Chevalley et al (2011)^b^ Gabel et al (2017b)^a^ Mitchell et al (2018)^b^
Foot bones	Figus et al (2022) ^b^ Saers et al (2020) ^a^	Figus et al (2022) ^b^ Saers et al (2020) ^a^	Figus et al (2022) ^b^ Saers et al (2020) ^a^	Figus et al (2022) ^b^ Saers et al (2020) ^a^	Saers et al (2020) ^a^

a Only BV/TV values provided in the reference were adopted for the present study.

b Both BV/TV and Tb.N values provided in the reference were adopted for the present study.

c These references provided identical datasets.

**Table 3.** Target trabecular number (Tb.N), bone volume fraction (BV/TV), and cellularity factor (CF) established for the detailed pediatric skeletal models, along with the scaling factor (SF) for achieving the target Tb.N.

		Newborn			1 yr-old			5 yr-old			10 yr-old			15 yr-old male			15 yr-old female		
Skeletal site of *μ*CT images	Target skeletal site	Target Tb.N (SF)	Target BV/TV	Target CF	Target Tb.N (SF)	Target BV/TV	Target CF	Target Tb.N (SF)	Target BV/TV	Target CF	Target Tb.N (SF)	Target BV/TV	Target CF	Target Tb.N (SF)	Target BV/TV	Target CF	Target Tb.N (SF)	Target BV/TV	Target CF
Frontal bone	Frontal bone	2.07 (1.00)	0.65	1.00	2.07 (1.00)	0.76	0.97	2.07 (1.00)	0.66	0.84	2.07 (1.00)	0.48	0.68	2.07 (1.00)	0.37	0.59	2.07 (1.00)	0.37	0.58
Parietal bone	Parietal bone	2.19 (1.00)	0.65	1.00	2.19 (1.00)	0.76	0.97	2.19 (1.00)	0.66	0.84	2.19 (1.00)	0.48	0.68	2.19 (1.00)	0.37	0.59	2.19 (1.00)	0.37	0.58
Occipital bone	Occipital bone	2.00 (1.00)	0.65	1.00	2.00 (1.00)	0.76	0.97	2.00 (1.00)	0.66	0.84	2.00 (1.00)	0.48	0.68	2.00 (1.00)	0.37	0.59	2.00 (1.00)	0.37	0.58
Mandible	Mandible	2.87 (0.40)	0.47	1.00	1.98 (0.58)	0.35	0.97	1.43 (0.80)	0.29	0.84	1.33 (0.86)	0.24	0.68	1.42 (0.81)	0.20	0.59	1.32 (0.87)	0.19	0.58
Scapulae	Scapulae	1.64 (0.66)	0.45	1.00	1.98 (0.55)	0.35	0.97	1.43 (0.76)	0.29	0.84	1.33 (0.82)	0.24	0.68	1.42 (0.76)	0.20	0.59	1.32 (0.83)	0.19	0.58
Clavicles	Clavicles	2.87 (0.30)	0.47	1.00	1.98 (0.44)	0.35	0.97	1.43 (0.60)	0.29	0.83	1.33 (0.65)	0.24	0.66	1.42 (0.61)	0.20	0.56	1.32 (0.66)	0.19	0.55
Sternum	Sternum	2.87 (0.44)	0.51	1.00	1.98 (0.63)	0.35	0.97	1.43 (0.88)	0.29	0.89	1.33 (0.94)	0.24	0.83	1.42 (0.88)	0.20	0.81	1.32 (0.95)	0.19	0.79
Upper ribs	R1–R4	1.76 (0.47)	0.37	1.00	1.45 (0.57)	0.40	0.97	1.12 (0.74)	0.26	0.89	0.74 (1.11)	0.13	0.83	1.92 (0.43)	0.22	0.81	1.60 (0.52)	0.17	0.79
Middle ribs	R5–R8	1.76 (0.76)	0.37	1.00	1.45 (0.93)	0.40	0.97	1.12 (1.20)	0.26	0.89	0.74 (1.80)	0.13	0.83	1.92 (0.70)	0.22	0.81	1.60 (0.84)	0.17	0.79
Lower ribs	R9–R12	1.76 (0.79)	0.37	1.00	1.45 (0.96)	0.40	0.97	1.12 (1.23)	0.26	0.89	0.74 (1.86)	0.13	0.83	1.92 (0.72)	0.22	0.81	1.60 (0.87)	0.17	0.79
C3	C1–C3	2.21 (1.00)	0.78	1.00	1.23 (1.80)	0.30	0.97	1.22 (1.81)	0.26	0.89	1.21 (1.82)	0.19	0.83	1.22 (1.80)	0.15	0.81	1.20 (1.84)	0.15	0.79
C6	C4–C7	2.21 (0.78)	0.78	1.00	1.23 (1.40)	0.30	0.97	1.22 (1.41)	0.26	0.89	1.21 (1.42)	0.19	0.83	1.22 (1.40)	0.15	0.81	1.20 (1.44)	0.15	0.79
T1	T1–T2	2.17 (0.84)	0.74	1.00	0.93 (1.96)	0.20	0.97	0.92 (1.97)	0.18	0.89	0.92 (1.98)	0.13	0.83	0.91 (2.01)	0.10	0.81	0.91 (1.99)	0.10	0.79
T3	T3–T4	2.17 (0.63)	0.74	1.00	0.93 (1.48)	0.20	0.97	0.92 (1.49)	0.18	0.89	0.92 (1.50)	0.13	0.83	0.91 (1.52)	0.10	0.81	0.91 (1.51)	0.10	0.79
T6	T5–T7	2.17 (0.52)	0.74	1.00	0.93 (1.22)	0.20	0.97	0.92 (1.22)	0.18	0.89	0.92 (1.23)	0.13	0.83	0.91 (1.25)	0.10	0.81	0.91 (1.24)	0.10	0.79
T9	T8–T10	2.17 (0.76)	0.74	1.00	0.93 (1.79)	0.20	0.97	0.92 (1.80)	0.18	0.89	0.92 (1.81)	0.13	0.83	0.91 (1.84)	0.10	0.81	0.91 (1.82)	0.10	0.79
T11	T11	2.17 (0.65)	0.74	1.00	0.93 (1.51)	0.20	0.97	0.92 (1.52)	0.18	0.89	0.92 (1.53)	0.13	0.83	0.91 (1.55)	0.10	0.81	0.91 (1.54)	0.10	0.79
T12	T12	2.17 (0.60)	0.74	1.00	0.93 (1.41)	0.20	0.97	0.92 (1.42)	0.18	0.89	0.92 (1.43)	0.13	0.83	0.91 (1.44)	0.10	0.81	0.91 (1.43)	0.10	0.79
L1	L1	2.34 (0.52)	0.63	1.00	0.78 (1.57)	0.18	0.97	1.49 (0.82)	0.17	0.89	1.43 (0.85)	0.14	0.83	1.38 (0.89)	0.11	0.81	1.38 (0.89)	0.11	0.79
L2	L2	2.34 (0.55)	0.63	1.00	0.78 (1.65)	0.18	0.97	1.49 (0.87)	0.17	0.89	1.43 (0.90)	0.14	0.83	1.38 (0.94)	0.11	0.81	1.38 (0.94)	0.11	0.79
L3	L3	2.34 (0.59)	0.63	1.00	0.78 (1.76)	0.18	0.97	1.49 (0.93)	0.17	0.89	1.43 (0.96)	0.14	0.83	1.38 (1.00)	0.11	0.81	1.38 (1.00)	0.11	0.79
L4	L4	2.34 (0.49)	0.63	1.00	0.78 (1.48)	0.18	0.97	1.49 (0.78)	0.17	0.89	1.43 (0.81)	0.14	0.83	1.38 (0.84)	0.11	0.81	1.38 (0.84)	0.11	0.79
L5	L5	2.34 (0.67)	0.63	1.00	0.78 (2.02)	0.18	0.97	1.49 (1.06)	0.17	0.89	1.43 (1.10)	0.14	0.83	1.38 (1.14)	0.11	0.81	1.38 (1.14)	0.11	0.79
Sacrum	Sacrum	2.40 (0.56)	0.55	1.00	1.08 (1.23)	0.51	0.97	0.90 (1.49)	0.43	0.89	0.96 (1.39)	0.32	0.83	1.03 (1.30)	0.25	0.81	1.03 (1.30)	0.25	0.79
Os coxae	Os coxae	2.00 (0.75)	0.42	1.00	2.14 (0.70)	0.46	0.97	1.71 (0.87)	0.23	0.83	1.79 (0.83)	0.23	0.75	1.64 (0.91)	0.19	0.69	1.64 (0.91)	0.19	0.67
Proximal humeri	Proximal humeri	2.72 (0.40)	0.34	1.00	1.51 (0.71)	0.30	0.97	1.22 (0.88)	0.19	0.81	1.12 (0.96)	0.19	0.62	1.14 (0.94)	0.12	0.48	1.14 (0.94)	0.12	0.47
Distal humeri	Distal humeri	2.82 (0.62)	0.40	1.00	1.54 (1.14)	0.17	0.91	1.29 (1.35)	0.24	0.74	1.26 (1.38)	0.17	0.41	1.26 (1.39)	0.14	0.11	1.21 (1.44)	0.13	0.11
	Hand bones	2.87 (0.61)	0.47	1.00	1.98 (0.88)	0.35	0.51	1.43 (1.22)	0.29	0.21	1.33 (1.31)	0.24	0.00	1.42 (1.23)	0.20	0.00	1.32 (1.33)	0.19	0.00
Proximal radii	Proximal radii	3.01 (0.26)	0.46	1.00	2.44 (0.32)	0.33	0.91	1.38 (0.56)	0.22	0.60	1.24 (0.62)	0.22	0.24	1.04 (0.75)	0.20	0.00	1.04 (0.75)	0.20	0.00
Distal radii	Distal radii	4.46 (0.33)	0.29	1.00	3.61 (0.41)	0.21	0.91	2.04 (0.72)	0.14	0.60	1.84 (0.80)	0.13	0.24	2.01 (0.73)	0.12	0.00	1.79 (0.82)	0.11	0.00
Proximal ulnae	Proximal ulnae	3.01 (0.45)	0.46	1.00	2.44 (0.55)	0.33	0.91	1.38 (0.98)	0.22	0.60	1.24 (1.09)	0.22	0.24	1.04 (1.30)	0.20	0.00	1.04 (1.30)	0.20	0.00
Distal ulnae	Distal ulnae	4.46 (0.38)	0.29	1.00	3.61 (0.48)	0.21	0.91	2.04 (0.84)	0.14	0.60	1.84 (0.93)	0.13	0.24	2.01 (0.85)	0.12	0.00	1.79 (0.96)	0.11	0.00
Femoral head	Femoral head	3.73 (0.50)	0.49	1.00	1.61 (1.15)	0.33	0.97	1.71 (1.08)	0.38	0.81	1.57 (1.18)	0.36	0.62	1.56 (1.19)	0.26	0.48	1.56 (1.19)	0.27	0.47
Femoral neck	Femoral neck	3.73 (0.37)	0.49	1.00	1.61 (0.87)	0.33	0.97	1.71 (0.81)	0.38	0.81	1.57 (0.88)	0.36	0.62	1.53 (0.91)	0.27	0.48	1.52 (0.91)	0.26	0.47
Distal femora	Distal femora	2.82 (0.71)	0.40	1.00	1.54 (1.31)	0.17	0.91	1.29 (1.56)	0.24	0.74	1.26 (1.59)	0.17	0.41	1.26 (1.60)	0.14	0.11	1.21 (1.66)	0.13	0.11
	Foot bones	1.79 (1.12)	0.24	1.00	1.37 (1.47)	0.27	0.51	1.17 (1.72)	0.24	0.21	1.19 (1.70)	0.28	0.00	1.18 (1.70)	0.23	0.00	1.18 (1.70)	0.23	0.00
Patellae	Patellae	2.87 (0.64)	0.47	1.00	1.98 (0.93)	0.35	0.91	1.43 (1.29)	0.29	0.60	1.33 (1.39)	0.24	0.24	1.42 (1.30)	0.20	0.00	1.32 (1.41)	0.19	0.00
Proximal tibiae	Proximal tibiae	3.01 (0.48)	0.46	1.00	2.44 (0.59)	0.33	0.91	1.38 (1.04)	0.22	0.60	1.24 (1.15)	0.22	0.24	1.04 (1.38)	0.20	0.00	1.04 (1.38)	0.20	0.00
Distal tibiae	Distal tibiae	3.59 (0.50)	0.28	1.00	2.90 (0.62)	0.20	0.91	1.64 (1.09)	0.14	0.60	1.48 (1.21)	0.13	0.24	2.13 (0.84)	0.12	0.00	1.50 (1.20)	0.11	0.00
Proximal fibulae	Proximal fibulae	3.01 (0.40)	0.46	1.00	2.44 (0.50)	0.33	0.91	1.38 (0.88)	0.22	0.60	1.24 (0.97)	0.22	0.24	1.04 (1.16)	0.20	0.00	1.04 (1.16)	0.20	0.00
Distal fibulae	Distal fibulae	3.59 (0.44)	0.28	1.00	2.90 (0.54)	0.20	0.91	1.64 (0.96)	0.14	0.60	1.48 (1.06)	0.13	0.24	2.13 (0.74)	0.12	0.00	1.50 (1.05)	0.11	0.00

**Table 4.** PHITS Monte Carlo radiation transport code environments for microscale and macroscale dosimetry simulations.

Item	Microscale dosimetry	Macroscale dosimetry
Code and version	PHITS version 3.35	
Model implementation	LAT = 3 in the [Cell] section (tetrahedral mesh geometry imported)	
Source generation	S-type = 17 in the [Source] section (pre-generated dump file used for computational efficiency, with source points generated using a barycentric coordinate–based method (Rocchini and Cignoni 2000))	s-type = 24 in the [Source] section (source generated within the tetrahedral mesh geometry)
Incident particle and energy	Proj = electron, e0 = set to 26 discrete values from 0.01 to 10, and dir = all in the [Source] section(monoenergetic electrons generated at discrete energy points between 0.01 and 10 MeV with isotropic emission)	
Reflective boundary (infinite geometry)	* in the [Surface] section (*-marked surface defined as a reflective boundary)	Not applied
History numbers	Maxcas = 10 000 and maxbch = 10 in the [Parameters] section (100 000 primary electrons transported)	
Physics	Negs = 1 in the [Parameters] section (EGS5 mode activated)	
Secondary cut value	emin(12,13) = 0.001 and emin(14) = 0.001 in the [Parameters] section (1 keV applied for electrons, positrons, and photons)	
Variance reduction	Not applied	
Scoring	Unit = 2 in the [T-Deposit] section (energy deposition [MeV/source] in the target region scored)	
Post-processing	Scored energy was divided by the primary electron energy to calculate the absorbed fraction (AF); the AF was further divided by the target region mass to obtain the specific absorbed fraction (SAF)	

**Table 5.** Comparison of resulting skeletal tissue masses obtained by integrating the detailed pediatric skeletal models into the mesh-type reference computational phantoms (MRCPs) of ICRP Publication 156 (2024), with the corresponding ICRP target masses (2002, 2024).

Skeletal tissue	Newborn			1 yr-old			5 yr-old		
	Target (g)	Result (g)	Diff. (%)	Target (g)	Result (g)	Diff. (%)	Target (g)	Result (g)	Diff. (%)
Trabecular bone	119.0	119.7	0.6	362.0	356.5	1.5	622.4	615.5	1.1
RBM	71.6	71.1	0.7	195.2	198.9	1.9	464.8	468.7	0.8
YBM	—	—	—	15.9	15.8	0.3	132.5	133.1	0.5
	10 yr-old			15 yr-old male			15 yr-old female		
Skeletal tissue	Target (g)	Result (g)	Diff. (%)	Target (g)	Result (g)	Diff. (%)	Target (g)	Result (g)	Diff. (%)

Trabecular bone	753.7	753.9	0.0	1077.1	1075.5	0.1	952.9	951.2	0.2
RBM	902.6	893.5	1.0	1317.2	1312.5	0.4	1257.8	1254.8	0.2
YBM	531.2	539.8	1.6	1579.6	1585.9	0.4	1393.2	1397.1	0.3

**Table 6.** Masses of red bone marrow (RBM) and endosteum obtained by integrating the pediatric skeletal models into the mesh-type reference computational phantoms (MRCPs) of ICRP Publication 156 (ICRP 2024).

Skeletal site (Spongiosa)	Newborn		1 yr-old		5 yr-old		10 yr-old		15 yr-old male		15 yr-old female	
	RBM (g)	Endo. (g)	RBM (g)	Endo. (g)	RBM (g)	Endo. (g)	RBM (g)	Endo. (g)	RBM (g)	Endo. (g)	RBM (g)	Endo. (g)
Cranium	20.608	14.735	52.765	43.197	120.555	99.453	140.477	119.980	156.004	132.456	119.896	104.852
Mandible	2.545	1.912	6.195	3.183	14.084	5.490	11.887	4.879	15.289	7.158	11.539	5.007
Scapulae	2.058	0.928	7.706	3.806	21.480	8.292	40.972	17.068	44.019	21.242	57.936	25.914
Clavicles	0.751	0.456	1.012	0.415	4.025	1.326	7.709	2.831	8.645	3.680	10.672	4.275
Sternum	0.281	0.224	1.116	0.579	4.858	1.795	12.427	4.236	25.773	9.039	17.256	5.524
Ribs	11.544	4.508	28.869	9.768	44.212	10.468	91.469	10.892	115.554	50.428	126.789	43.114
Cervical vertebrae	1.220	1.004	5.399	1.399	7.867	2.067	18.599	4.527	35.336	7.998	42.087	9.369
Thoracic vertebrae	2.128	1.737	19.694	3.490	48.486	8.820	141.314	23.693	178.198	27.289	151.944	23.901
Lumbar vertebrae	2.340	1.803	14.557	2.041	37.170	11.339	103.926	29.633	172.233	44.622	178.727	47.153
Sacrum	1.151	0.841	6.073	2.099	15.882	4.538	30.323	8.274	69.513	18.823	80.619	22.235
Os coxae	4.233	2.228	15.891	9.398	55.932	22.559	150.729	70.926	269.732	114.607	287.375	123.540
Proximal humeri	1.890	1.266	4.618	1.808	10.826	3.403	22.682	8.438	68.071	28.184	51.581	21.768
Distal humeri	1.360	0.974	2.632	0.803	5.164	1.808	7.581	4.175	6.414	10.902	5.015	8.065
Proximal radii	0.270	0.145	0.340	0.198	0.422	0.251	0.876	1.120	—	4.890	—	1.779
Distal radii	0.626	0.592	0.208	0.167	0.991	0.548	0.399	0.459	—	2.985	—	3.997
Proximal ulnae	0.628	0.495	0.791	0.483	1.858	0.813	1.779	1.724	—	5.836	—	4.545
Distal ulnae	0.418	0.389	0.029	0.024	0.254	0.144	0.213	0.251	—	2.095	—	1.232
Hand bones	1.440	1.116	3.745	3.427	2.087	3.043	—	6.768	—	12.034	—	8.959
Proximal femora	2.362	2.173	4.605	1.784	10.787	5.623	25.035	15.430	80.008	53.623	57.588	39.638
Distal femora	2.341	1.702	5.309	1.667	13.778	4.943	25.901	14.817	27.474	47.460	24.311	40.003
Patellae	0.077	0.063	0.292	0.164	2.232	1.232	2.265	2.610	—	4.660	—	3.390
Proximal tibiae	1.801	1.490	2.884	1.856	7.110	3.059	8.145	7.684	—	25.580	—	21.896
Distal tibiae	1.468	1.217	0.674	0.427	2.061	0.839	2.271	1.962	—	17.068	—	10.351
Proximal fibulae	0.237	0.180	0.149	0.092	0.565	0.252	0.661	0.644	—	2.232	—	1.658
Distal fibulae	0.494	0.384	0.142	0.094	0.637	0.292	0.659	0.643	—	4.095	—	2.178
Foot bones	3.221	1.207	6.245	3.589	9.400	11.011	—	27.108	—	69.074	—	54.696
Humeri (upper shaft)	0.322	0.019	0.809	0.052	2.959	0.133	5.338	0.248	7.782	0.367	5.917	0.322
Humeri (lower shaft)	0.322	0.020	0.744	0.050	2.737	0.123	5.227	0.240	3.947	0.352	2.952	0.296
Radii	0.122	0.014	0.326	0.041	1.421	0.129	1.656	0.250	—	0.272	—	0.255
Ulnae	0.143	0.016	0.535	0.055	1.629	0.136	1.816	0.257	—	0.307	—	0.284
Femora (upper shaft)	0.737	0.034	0.939	0.059	6.466	0.254	13.728	0.502	19.863	0.796	13.736	0.597
Femora (lower shaft)	1.174	0.048	1.448	0.076	4.458	0.157	9.045	0.331	8.636	0.511	8.870	0.602
Tibiae	0.673	0.041	1.996	0.121	5.123	0.290	6.920	0.591	—	1.003	—	0.921
Fibulae	0.107	0.014	0.183	0.036	1.160	0.133	1.470	0.264	—	0.353	—	0.299

Total	71.1	44.0	198.9	96.4	468.7	214.8	893.5	393.5	1312.5	732.0	1254.8	642.6

## Data Availability

All data that support the findings of this study are included within the article (and any supplementary information files).
